# Spousal Care Intensity, Socioeconomic Status, and Depression among the Older Caregivers in China: A Study on 2011–2018 CHARLS Panel Data

**DOI:** 10.3390/healthcare10020239

**Published:** 2022-01-26

**Authors:** Jun Ma, Hongyan Yang, Wenxiu Hu, Hafiz T. A. Khan

**Affiliations:** 1 Center for Social Security Studies, Wuhan University, Wuhan 430072, China; xtmajun@whu.edu.cn; 2 Center for Population and Development Policy Studies, Fudan University, Shanghai 200433, China; wenxiu_hu@fudan.edu.cn; 3 Postdoctoral Research Workstation, China Everbright Group, Beijing 100033, China; 4 Public Health Group, College of Nursing, Midwifery and Healthcare, University of West London, Brentford, London TW8 9GB, UK; hafiz.khan@uwl.ac.uk

**Keywords:** Chinese older adults, spousal caregivers, care intensity, depression, socioeconomic status

## Abstract

Using the stress process model and data from the 2011–2018 China Health and Retirement Longitudinal Study (CHARLS), this study examined the effect of spousal caregiving intensity on the depression level of older caregivers in China. The moderating role that socioeconomic status plays in the relationship between spouses was explored by constructing multilevel growth models (MGMs). The care intensity for a spouse was found to relate to significantly increased depression levels in older caregivers, while the degree of disability of the spouse being cared for (B = 0.200, *p* < 0.001) having a greater effect on depression than the duration of care (B = 0.007, *p* < 0.01). There was a threshold effect where the provision of more than 10 h of care per week for a spouse (B = 0.931, *p* < 0.001; B = 0.970; *p* < 0.01) or caring for a disabled spouse with limited ADLs (B = 0.709, *p* < 0.01; B = 1.326; *p* < 0.001; B = 1.469, *p* < 0.01) increased depression in older caregivers. There were moderating influences, including higher professional prestige before retirement (B = −0.006, *p* < 0.05) and higher annual family income (B = −0.037, *p* < 0.10), that increased depression related to the spouse’s degree of disability. It was considered that active familism measures should be formulated for older spousal caregivers, especially those with lower socioeconomic status.

## 1. Introduction

The stress of caring for older adults within the context of population aging has become a major factor affecting the health of caregivers. The average time that elders in China receive care is around 4–8 years [1]. In EU countries, life expectancy after reaching the age of 65 is estimated to be 18 years for men and 22 years for women. However, healthy life years after 65 are about 10 years for both genders [2]. At the global level, home has always been an important place for disabled older adults to receive care. This is due to the scarcity of formal care resources, the high cost, and the preference of older adults for continuing to live at home. It is estimated that approximately 70–90% of caregivers in OECD countries are informal family caregivers [3,4]. In China, due to the influence of social customs and filial values, as well as the promotion of the Marriage Law and Law on Protection of the Rights and Interests of the Elderly, caring for older adults is considered an obligation of family members [5].

However, with the outflow of the youth labor force, a rise in the number of nuclear families, and increases in female employment rates, spouses are increasingly taking on the role of caregivers of disabled older adults within families. The theory of the deinstitutionalization of marriage argues that the meaning of marriage in contemporary times has changed from institutional to companionate marriage [6]. Studies in mainland China, Hong Kong, and Japan have shown that spousal caregivers account for about 30% of all family caregivers and are most likely to assume the primary caregiving role even when living with their children [7,8,9]. Due to feelings of mutual support and gratitude, spouses are often able to provide the most selfless and attentive care. However, this “labor of love” [10], as Graham calls it, is time-consuming, stressful, difficult, and demanding. Since spousal caregivers are most likely to live with disabled older adults for long periods of time, they tend to spend more time on care and have less respite than their children or other caregivers [11]. When the physiological functions of older adults are in decline, the role of spousal caregivers can lead to issues such as great mental stress and health burden. Paying attention to the depression levels of spousal caregivers is significant in a number of ways: for helping to postpone the time when disabled older adults may need to move into care institutions; for controlling medical costs; for protecting the mental health of caregivers; and for improving the quality of care.

Since 1970, gerontology and psychology in the West have focused on the impact of caregiving on depression in the family. The common strategy was to compare whether there was a significant difference in depression levels between caregivers and non-caregivers, but research findings have been inconsistent. On the one hand, some findings suggested that family caregiving activities contained factors that had a positive impact on mental health, such as gaining greater satisfaction and accomplishment [12], having a meaningful life [13], and enhancing the relationship between the caregivers and care recipients [14], thus reducing depression levels. On the other hand, there were suggestions that caregiving activities were demanding and stressful and could significantly increase the incidence rates of psychological disorders such as depression and anxiety [15,16,17], leading to sleep disorders [18], endocrine disorders [19], and greater medical needs [20]. The impact of depression on family caregivers therefore needed to be reexamined. The cumulative consistent evidence showed that 20–30% of informal caregivers for elderly cancer patients may have a high risk of developing psychological disorders such as depression [21]. A survey by the Family Caregiver Alliance conducted in the U.S. showed that 40–70% of caregivers expressed clinically significant symptoms, with one-fourth to one-half of them meeting the diagnostic criteria for major depressive disorder [22]. One study in South Australia showed that 19.1% of female spousal caregivers of elderly cancer survivors had moderate depression and 23.6% had severe depression [23]. Also, spousal caregivers were 2.51 times more likely to suffer from depression than non-spousal caregivers of patients with Alzheimer’s Disease (AD) and to have higher levels of depression than parent caregivers and daughter or daughter-in-law caregivers [24,25,26].

Several studies focused on the impact of care intensity on depression in family caregivers from the perspectives of both the caregiver and care recipient. There were disagreements among research findings related to the effect of the time devoted to caregiving on depression levels in caregivers. Using the Patient Health Questionnaire-9 and multiple linear regression, one study in Canada showed that more hours spent on weekly caregiving were associated with more pronounced depressive symptoms [27]. Another study in Ethiopia using logistic regression analysis showed that caregivers of patients with mental illness who provided care for more than 6 h per day were at a significantly increased risk of depression [28]. However, one study in Poland used the Center for Epidemiological Studies Depression Scale (CES-D) and the Spearman’s rank correlation coefficient and found no significant correlation between the number of hours per week spent on caring for a patient with dementia and the severity of the caregiver’s depression [29]. Other studies in China, Korea, and Japan discovered that caregivers caring for family members living with dementia or requiring ADL assistance tended to have higher symptoms of depression than those caring for recipients that could manage daily activities [30,31,32].

A caregiver’s reaction to stress is not always negative and indeed, could serve to mobilize their own resources to seek countermeasures to help alleviate any stress and depression. The socioeconomic status of caregivers has received some attention in the literature. For example, regarding education, a study in the U.S. found that caregivers with lower-level education experienced more stress that may be related to their lack of knowledge and access to information about health care support [33]. One study, based on a sample from Shanghai, China, found that the education levels of caregivers who cared for older adults with a functional disability had significant moderating effects on the correlation between the ADLs (activities of daily living) of the older adults being cared for and family caregiver burden [34]. When compared to American family and friend caregivers with low economic vulnerability, those with high economic vulnerability were 100% more likely to experience severe emotional distress [35]. Based on the caregiving stress model, caregivers that had a poor financial status and cared for dementia patients were less aware of the poor physical and mental health of the patients and hence less likely to receive health care services or support [36]. The better-off caregivers may have had more financial resources to purchase professional services to meet the care needs of their relatives, thereby enhancing the well-being of such caregivers [37]. Occupational factors may have played a role here. By using national longitudinal data from the U.S. Health and Retirement Study and the multivariate regression models, one exploratory research project showed that employed informal caregivers had significantly higher levels of depression than retired informal caregivers [38].

While previous studies explored the impact of caregiving on the depression levels of caregivers, the main purpose of this study was to investigate the impact of older spousal caregiving on depression. It focused on the heterogeneity of the effects of caregiving intensity, and further explored the moderating effect of socioeconomic status responsible for depression. The unique approach of this study can be seen in the following three points: first, unlike most previous studies that were based on regional and cross-sectional data, this study aimed to assess the effect of care intensity on the depression levels of older caregivers by using national tracking data and constructing multilevel growth models (MGMs); second, previous studies focused on the caregivers of people with specific medical conditions such as dementia and stroke, with little attention paid to older spousal caregivers in China; third, previous studies treated caregivers as a homogeneous group, and the intensity of caregiving stressors was usually ignored, whereas this study focused on the intensity measures of caregiving from the perspectives of both the caregiver and care recipient. The two intensity measures, duration of care and the degree of disability of the spouse being cared for, were explored and compared to determine which of them had a greater impact on the depression degree among older spousal caregivers. An answer was sought as to what heterogeneity existed in the effects of caregiving intensity on spousal caregiver depression levels and whether there was a threshold for the time devoted to caregiving and a threshold for the level of disability of the care recipient that could affect the depression degree. Finally, the moderating effects of socioeconomic status such as education, occupational prestige, and household income were comprehensively examined.

## 2. Theoretical Basis and Hypotheses

The stress process model provided the theoretical foundation for this study. The stress process consists of three components: stressors, moderators, and outcomes [39]. Stressors refer to the experience and environment that generate stress, either from acute life events or chronic life stress. Moderators refer to the individual’s response to stress by positional advantage, social support, or coping strategies adopted to prevent or mitigate the harm caused by the stressor. Effective stress coping resources are unequally distributed in society, with males, the educated, and the wealthy able to cope more effectively with stress. Outcomes refer to the performance of the body’s response to stress that exists in many manifestations, such as the number and extent of chronic illnesses and the probability of mental illness.

The theory can be developed into two hypotheses, where the first hypothesis relates to stress exposure. When individuals are exposed to different stressful situations, the impact on health outcomes varies. For example, when the stress of a social role exceeds an individual’s physical and psychological capacity, it can become a stressor that is detrimental to health. Caregivers can be subjected to chronic life stress over long periods of time, such as the 3–15 years of average caregiving responsibilities for those looking after individuals with dementia. Caregivers have to continually monitor and witness the decline in the self-care abilities of progressive dementia patients and this can lead to psychological problems [40]. Depressive symptoms caused by caregiving may be more pronounced for older caregivers that have less respite and those who care for spouses with higher levels of disability. Therefore, hypothesis one is as follows:

- Depression in older spousal caregivers is influenced by caregiving stressors. There is no threshold of care intensity that affects depression levels. The longer the time spent on caring for a spouse and the higher the level of disability of the spouse being cared for, then the higher the level of depression will be in the older caregivers.

**Figure 1:**
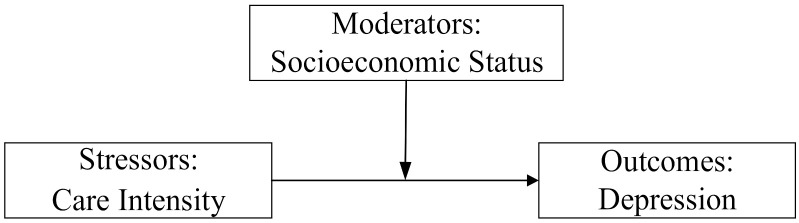
The moderating effect of socioeconomic status.

The second hypothesis concerns stress vulnerability. When trying to cope with stress, an individual’s vulnerability due to low social status, lack of resources, and coping strategies are the main reasons for the differences in health outcomes. People with lower socioeconomic status are at a disadvantage in terms of mobilizing material and psychosocial resources, stress relief and risk perception, for instance, thus showing greater vulnerability in their responses to stress. In addition, the accumulation hypothesis suggests that the health disadvantages/advantages of individuals with a lower/higher socioeconomic status due to a lack/sufficiency of resources will accumulate with age [41]. The impact of socioeconomic status on health will be amplified in old age. Therefore, hypothesis two is as follows:

- The effects of caregiving stressors on depression in spousal caregivers are moderated by the caregiver’s socioeconomic status (Figure 1). The higher the socioeconomic status of an individual, the less likely the intensity of spousal caregiving will influence the level of a caregiver’s depression.

## 3. Materials and Methods

### 3.1. Data Source and Study Population

Data were derived from the China Health and Retirement Longitudinal Study (CHARLS). This large-scale interdisciplinary survey project hosted by The National School of Development (NSD) at Peking University aims to collect a set of high-quality microdata representing families and individuals aged 45 and older in China. The CHARLS questionnaire included the following modules: demographics, family structure/transfer, health status, work and retirement, and income and consumption, among others. The baseline national wave of CHARLS was completed in 2011 and followed up in 2013, 2015, and 2018 and included around 17,500 individuals in 150 counties/districts and 450 villages/resident committees. CHARLS collected data covering a total of 12,400 households and 19,000 individuals by 2018. It used sampling based on multi-stage stratified probabilities proportional to size (PPS), and the development of an innovative software package called CHARLS-GIS helped to produce village sampling frames. A supervisor randomly sampled 80 households in each community/village and within those households, the family member over 45 years of age and his/her spouse were randomly selected as the main respondents.

The sample in this study consisted of both partners and spouses in the household and included four waves of CHARLS from 2011–2018. This study limited the sample to older adults who were aged 60 years and older, could be matched with the sample of older spouses, and had fully answered key questions such as depression symptoms in the baseline wave and completed at least one follow-up survey. The sample sizes of the four waves finally included in this study were 1511, 1511, 1228, and 876, respectively.

### 3.2. Measures

#### 3.2.1. Depression

The dependent variable of “depression” in this study was measured using the Center for Epidemiologic Studies Depression Scale-10 (CESD-10) that was developed by Andresen et al. (1994) [42]. The scale asks respondents 10 questions on how they felt and behaved during the last week, including the following: I was bothered by small things; I had trouble keeping my mind on what I was doing; I felt depressed; I felt everything I did was an effort; I felt hopeful about the future; I felt fearful; my sleep was restless; I was happy; I felt lonely; I felt I could not get “going”. The response options range from rarely or none of the time (<1 day) = 0, some or a little of the time (1–2 days) = 1, occasionally or a moderate amount of the time (3–4 days) = 2, and most or all of the time (5–7 days) = 3. The two positive statements “I felt hopeful about the future” and “I was happy” were reverse scored to obtain the total score of depressive symptoms; the higher the score, the more severe the depressive symptoms. Based on Andresen’s criteria, a CESD-10 score of ≥10 was considered as depression. As shown in Table 1 the mean score of the older adults in the sample was close to the threshold value, and the mental health status was not optimistic.

#### 3.2.2. Care Intensity

The core independent variable in this study was “care intensity”, measured by the weekly caregiving duration and the degree of disability of the spouse being cared for. The two indicators reflected the caregiver’s involvement and the care recipient’s needs. Previous studies had also examined these two indicators when measuring care intensity [43,44,45]. The variables were set as continuous variables and further consideration was given to setting up categorical variables in order to examine in more detail the heterogeneity of care intensity on the level of depression among older caregivers.

In the time devoted to caregiving, some studies focused on a threshold of 10 h per week to distinguish between high and low levels of care [46,47].^,^ The research report of family older care policies in OECD countries in 2011 found that care intensity was close to 20 h per week [48], while some studies set the intensity threshold of the duration of care at 15 h per week [49,50]. In recent studies, duration of care has been defined more precisely. For example, one study analyzed the relationship between grandparent caregiving and depression levels, which set the intensity threshold at 0, 10, and 40 h per week [51]. The average number of hours that older caregivers spent on care for their spouses in the sample of the current study reached 34 h per week. Considering this finding, it may not be quite consistent with the actual situation presented by the data if the care intensity was classified into high and low intensity using only 10, 15, or 20 h per week as the threshold. Therefore, this study classified caregiver participation as no care participation (0 h per week), low-level (0.1–9.9 h per week), moderate-level (10–39.9 h per week), and high-level (40–168 h per week). As shown in Table 1, the number of hours that older caregivers spent on spousal care per week increased each year. Specifically, the proportion of older adults that did not participate in caregiving for their spouses decreased each year; the proportion of low-level care participation increased each year; the proportion of moderate-level care participation increased and then decreased; and the proportion of high-level care participation decreased and then increased.

In this study, older adults having difficulty with at least one of the ADLs (activities of daily living) were defined as disabled. The disability degree of the spouse being cared for was measured by the KATZ scale, asking older adults whether they need help with the six ADLs: bathing, dressing, eating, getting into or out of bed, using the toilet, and continence. Each item was rated as complete independence = 1, partial independence = 2, and complete dependence on others = 3. The scores were summed, and total scores ranged from 6 to 18. According to classification criteria in previous studies [52,53], the degree of disability of the spouse being cared for was classified as no disability (Note: The reason why some older adults being cared for in the sample are non-disabled is that CHARLS have some samples with unlimited ADLs but limited instrumental activities of daily living (IADLs), including doing household chores, cooking, shopping, taking medications, and money management.) (ADL = 6), mild disability (ADL = 7–10), moderate disability (ADL = 11–14), and severe disability (ADL = 15–18). As shown in Table 1, among the types of older adults involved in caregiving, those caring for spouses with a mild disability were the most numerous.

#### 3.2.3. Socioeconomic Status (SES)

The moderating variable “socioeconomic status” referred to the amount of power, resources, and opportunities that people could obtain due to their position in society and could greatly affect the resources provided to family members. The socioeconomic status of older adults can be measured primarily in terms of education level, professional prestige before retirement, and annual family income. Among these, professional prestige before retirement was based on the score obtained from Treiman’s Standard International Occupational Prestige Scale (SIOPS) [54]. In the study sample, except for the older adults that had never worked (occupational prestige value of 0), the minimum value of occupational prestige was 13, which corresponded to manual laborers such as garbage scavengers and cleaners, while the maximum value was 78, which corresponded to doctors and professional teaching staff in higher education. Annual family income consisted of five components in the questionnaire: wage income; personal transfer income such as pension and old age allowance; household agricultural income; household self-employment income; and household transfer income such as subsidies for returning farmland to forest and agricultural subsidies. The operationalization of the key concepts of the stress process model in this study is summarized in Table 2.

#### 3.2.4. Covariates

The covariates in this study consisted of four aspects: personal characteristics of caregivers, health behaviors, intergenerational support, and social support. Personal characteristics included age, gender, ADLs, and area; health behavioral factors included participation in social activities, smoking, and exercise; intergenerational support factors included the frequency of contact; and social support factors included pension and medical insurance participation.

### 3.3. Statistical Analysis

In the first instance, analysis of variance (ANOVA) was used to compare the differences in depression levels of older caregivers at different caregiving intensities. Secondly, the multilevel growth models (MGMs) of the hierarchical linear model (HLM) were used to study the effect of spousal caregiving intensity on the depression level of older caregivers and the moderating role of socioeconomic status in the relationship.

The idea of analyzing individual tracking data through the HLM was first introduced when Huttenlocher et al. (1991) collected tracking data from children in order to study individual vocabulary growth [55]. This statistical analysis technique is now widely used in various academic fields. Generally, in longitudinal data analysis, it is often required that all study subjects must be observed at every point in time. Once there are missing data, the samples with missing data must be excluded. In contrast, the MGM has relatively low requirements on raw data. It is suitable for analyzing longitudinal data with repeated observations on the same individual and with missing tracking times. The model does not have strict restrictions on either the number of repeated measurements or the time interval between repeated measurements. There are sample losses in the four-wave longitudinal data survey as individuals were not surveyed at every time point. Therefore, the MGM can be used for the sample size to the greatest extent and so reduce any bias of an estimator.

The level-1 model studied inter-individual variability in depression in older adults, that is, the effects of time-varying variables, such as care intensity, age, ADLs, annual family income, health behavior, intergenerational support, and social support. The level-2 model studied intra-individual variability, that is, the effects of variables that do not vary over time, such as gender, education level, professional prestige before retirement, and regional factors. Multiple measures for each individual (level-1) were considered as nested within the individual (level-2).

In the analysis process, a null model (Model 1) was constructed to judge the necessity of establishing an MGM that was based on the size of the intraclass correlation coefficient (ICC). The larger the ICC, the larger the variance in groups, and thus the greater the need to use MGM. In general, when ICC ≥ 0.059, it indicates that the between-group variances cannot be ignored, and the between-group effects must be considered in the MGM [56]; second, continuous variables (Model 2 and Model 3) and categorical variables (Model 4 and Model 5) of care intensity were added in turn to examine in-depth the heterogeneity of the effects of different care intensities on depression. The interaction variables of socioeconomic status and caregiving intensity (Model 6 and Model 7) were added to examine the moderating role of socioeconomic status factors in the relationship between spousal caregiving intensity and depression in older caregivers. In this study, HLM 6.08 software was used to estimate the models.

## 4. Results

First, we used ANOVA to make a preliminary comparison of depression in older caregivers at different levels of caregiving intensity. As shown in Table 3, the results showed that both the duration of care and the disability degree of the spouse being cared for had a significant effect on the depression level of older caregivers, with the disability degree of the spouse having a more significant effect on depression. The results showed overall that the longer the time spent on care and the higher the disability degree, the higher the depression level in the older caregivers.

The effects of different intensities of spousal care on depression among older caregivers were revealed by the results of the MGM (Table 4). The results of the null model for Model 1 showed that the value of ICC was 0.512, which was greater than 0.059. This indicated that the differences between groups could not be ignored and that an MGM was necessary. The results of Model 2 and Model 3 indicated that the longer the time spent on care for the spouse and the higher the disability degree of the spouse being cared for, the higher the level of depression of the older caregiver. In terms of the strength of the effect, the disability degree of the spouse had a greater impact on depression (B = 0.200, *p* < 0.001) than the duration of care (B = 0.007, *p* < 0.01).

Model 4 and Model 5 further examined the results for categorical variables. The effects of low-level care participation (B = 0.292, *p* > 0.05) and caring for a spouse without disability (with unlimited ADLs but limited IADLs) (B = 0.262, *p* > 0.05) on depression were not significant compared with older adults who had no care participation. Both moderate-level and high-level care participation (B = 0.931, *p* < 0.001; B = 0.970, *p* < 0.01) and caring for a disabled spouse (B = 0.709, *p* < 0.01; B = 1.326, *p* < 0.001; B = 1.469, *p* < 0.01) increased depression in caregivers. Hypothesis 1 was therefore partially confirmed.

As for covariates, the older the age, the higher the level of depression in older adults. Females had higher levels of depression than males. Depression levels were higher among those older adults who lived in rural areas, had a limited ability for self-care, did not receive intergenerational financial support, had infrequent contact with their children, and were not covered by pension plans. Depression levels were also higher among older adults with lower education levels and occupational prestige.

Table 5 examined the moderating role of socioeconomic status in the correlation between the intensity of spousal caregiving and depression. Socioeconomic status only moderated the relationship between the disability degree of the spouse and depression. It showed that the higher the professional prestige before retirement (B = 0.616, *p* < 0.01; B = −0.006, *p* < 0.05) and the higher the annual family income (B = 0.616, *p* < 0.01; B = −0.037, *p* < 0.10), the weaker effect of the disability degree in older adults on depression. Hypothesis 2 was therefore partially confirmed.

## 5. Discussion

Within families, spouses are increasingly taking on the role of caring for disabled older adults. Considering the paucity of research on the relationship between the intensity of spousal caregiving and depression among older caregivers in China, and especially the lack of longitudinal data studies based on nationally representative samples, this study set out to explore this association using data from a national survey sample conducted from 2011–2018.

First of all, this study found that the intensity of caring for a spouse significantly increased depression levels among older caregivers. According to the stress process model, caregivers viewed caregiving as a chronic stressor and a tedious task requiring high levels of commitment. Caregivers were vulnerable to great impacts in terms of time, physical strength, energy, and emotions and were prone to loneliness, anxiety, depression, and fatigue. As for the intensity of the effect, a key finding was that the disability degree of the spouse being cared for had a greater effect on depression than the duration of care. On the one hand, the reason for this may be that the spouse is usually the most important attachment figure for adults. Witnessing the spouse’s increasing level of disability, resulting in reduced mobility or being bedridden, could cause significant psychological stress to the spouse’s caregiver. On the other hand, some studies pointed out that the pathway of the effects of caregiving on significantly higher levels of depression involved a decrease in the caregiver’s ability to participate in the labor force and a reduction in their income [57]. However, for older caregivers, their time is of low economic value, and they will not endure as great an economic loss as their children, whose time will be more occupied. Therefore, care time has a relatively low impact on the increase in depression.

Second, the study further discovered that moderate-level and high-level intensity caregiving, as well as caring for a disabled spouse, increased depression. In contrast, low-level intensity care, that is, providing less than 10 h of care per week and caring for a non-disabled older adult with unlimited ADLs but limited IADLs, did not significantly increase depression levels in older caregivers. This finding suggested that there was a threshold effect in the impact of both the duration of care and the disability degree of the care recipient on the depression level of the spousal caregiver. This provided a further development and refinement of previous studies that concluded that the higher the care intensity, the more pronounced the caregiver’s depressive symptoms [27,28]. The stress process model suggests that when role overload or role strain exceeds an individual’s physical and psychological capacity, a chronic stressor that is harmful to health can develop [58]. Providing moderate care for spouses implies a marital commitment that could enhance the relationship of couples, instill a sense of accomplishment, and help caregivers find positive meaning in life. However, when the intensity of caregiving exceeds the point that caregivers can deal with, the expectations and responsibilities associated with the caregiving role can be very high, and this could interfere with daily life, recreation, and social interactions.

Third, for the moderating effect, it was found that socioeconomic status only moderated the relationship between a spouse’s disability degree and a caregiver’s depression level. It has been shown that higher professional prestige before retirement and higher annual family income were associated with weaker effects of the spouse’s disability degree on depression. The moderating effect of economic status has been confirmed by several studies [35,37]. Better household economic status indicates a greater ability to afford higher quality health care, and thus the disabled spouse can access and receive better care resources. Previous studies focused more on occupational prestige factors in the employed population. For example, one study found that higher occupational prestige reduced the prevalence of depression in the employed population [59]. The current study, however, found that professional prestige when employed continues to have a sustained and profound impact after retirement and can alleviate depressive symptoms in older spousal caregivers. One reason for this may be that older adults with higher professional prestige before retirement tend to have adequate socioeconomic and human capital. Their health advantages due to social resources also continue to accumulate as they age. As a result, they are able to cope better with stress and alleviate depressive symptoms.

In the 21st century, China has experienced rapid economic development, urbanization, accelerated population mobility, and the nuclearization of the family. Family values have been challenged in many aspects. For example, the family planning policy that has lasted for more than three decades has led to a large number of one-child families in China. Also, there have been a growing number of Chinese DINK (double income, no kids) families in recent years, and families have lost their dominant position in the construction of relationships. Spouses are playing an increasingly important role in caregiving and have become an important force in coping with the crisis of population aging. Chinese social security policies do not currently provide enough attention and support to family caregivers, which affects the welfare of the family and spousal caregivers and makes it challenging to ensure the quality of services received by older care recipients.

The government and society should take positive familism measures that reinforce family caregiving functions to mitigate the increasing effect of caregiving activities on the depression levels of older spousal caregivers. Firstly, support should be given to older spousal caregivers to balance their daily leisure time with their caregiving responsibilities and provide them with adequate respite. Drawing on the experience of Australia, diversified respite services, such as in-home day respite, in-home overnight respite, host family day respite, host family overnight respite, community-based day respite, community-based overnight respite, institution respite, and respite at emergency [60], can be provided to meet the different needs of caregivers. Secondly, caregiver organizations and groups should be created and the construction of information web platforms should be encouraged to provide reliable and convenient psychological counselling services, consultation, and training services, as well as information and coordination services. A number of nonprofit organizations for caregivers have been established in the United States, such as the Family Caregiver Alliance (FCA) and the National Family Caregivers Association. These organizations provide caregivers with direct support services and can intervene on caregiver burden issues. In addition, most of them have their own websites to provide caregivers with a range of online support resources. Mutual support groups for caregivers of special populations, such as groups for caregivers of people with dementia or chronic illnesses, could be established to connect with similar caregivers to share caregiving experiences and to receive advice and help. Again, a multi-level long-term care protection system should be established nationwide to provide financial compensation and to assure care for families of disabled older adults through social assistance or long-term care insurance. Given the scarcity of resources and the fact that older caregivers with higher socio-economic status have a stronger adjustment ability, the above family support policies should lean toward older spousal caregivers with low professional prestige before retirement and low family financial status.

There were two main limitations in this study. First, specific information was lacking about the details of caregiving activities in the CHARLS, for example, specific details of care provided, satisfaction of the spouse being cared for, spousal relationship, and subjective perceptions of caregiving stress that limited the ability to assess the effects of spousal caregiving intensity on depression levels in older caregivers. Second, subjective personal biases may have influenced answers as the data obtained were all from the subjective responses of Chinese older adults. Although depression is a common mental illness, Chinese people have negative attitudes toward people with mental illness, and stigmatization around it still exists. As a result, the participants might have provided socially acceptable responses and underestimated their own depression levels, thus leading to certain measurement errors. Since the survey did not consider social desirability biases, whether depression scores were underestimated or not could not be confirmed. It is expected that the above limitations could be overcome in future studies.

## 6. Conclusions

This study took the heterogeneity of care intensity into account. Using the 2011–2018 CHARLS panel data and MGM, the effects of spousal caregiving intensity on the depression level of older caregivers in China and the moderating role of socioeconomic status were examined. There were three main conclusions from this study: first, the intensity of caring for a spouse significantly increased depression levels in older caregivers, and the disability degree of the spouse being cared for had a greater effect on depression than the duration of care. Second, a key conclusion was that there was a threshold effect on the impact of the intensity of care on the depression level of the spousal caregiver, in that providing care for a spouse for more than 10 h per week or caring for a disabled spouse with limited ADLs increased depression. Third, socioeconomic status moderated the relationship between the disability degree of the spouse and depression, where higher professional prestige before retirement and higher annual family income were associated with weaker effects of the spouse’s disability degree on depression. The results showed that older spousal caregivers who took on high-level intensity caregiving in China had higher levels of depression, and their mental health status was not optimistic. Active familism measures should be developed and implemented for older spousal caregivers, especially those with low professional prestige before retirement and low family financial status, thus helping to prevent them from developing deep depression.

## Figures and Tables

**Table 1:** Descriptive statistics of the sample (2011–2018).

Variables	Measurement	Number (%)/Mean (SD)			
		2011 (*n* = 1511)	2013 (*n* = 1511)	2015 (*n* = 1228)	2018 (*n* = 876)
Dependent variable					
Depression	0–30, continuous measurement	9.63 (6.52)	8.69 (6.03)	9.13 (6.88)	9.55 (6.79)
Core independent variable: Care intensity					
Duration of care for spouses	0–168 h, continuous measurement	9.05 (27.04)	10.14 (23.51)	15.70 (37.71)	15.98 (37.31)
Disability degree of the spouse being cared for *	6–18, continuous measurement	9.34 (2.98)	8.94 (3.18)	9.32 (3.36)	9.31 (3.37)
Duration of care for spouses	No participation (0 h per week) = 0	1129 (74.72)	972 (64.33)	734 (59.77)	479 (54.68)
	Low-level care participation (0.1–9.9 h per week) = 1	134 (8.87)	143 (9.46)	189 (15.39)	183 (20. 89)
	Moderate-level care participation (10–39.9 h per week) = 2	149 (9.86)	327 (21.64)	176 (14.33)	119 (13.58)
	High-level care participation (40–168 h per week) = 3	99 (6.55)	69 (4.57)	129 (10.51)	95 (10.85)
Disability degree of the spouse being cared for	No participation = 0	1129 (74.72)	972 (64.33)	734 (59.77)	479 (54.68)
	No disability (unlimited ADL but limited IADL) (ADL = 6) = 1	70 (4.63)	153 (10.13)	121 (9.85)	92 (10.50)
	Mild disability (7–10) = 2	223 (14.76)	260 (17.21)	253 (20.61)	209 (23.86)
	Moderate disability (11–14) = 3	64 (4.24)	90 (5.95)	72 (5.86)	60 (6.85)
	severe disability (15–18) = 4	25 (1.65)	36 (2.38)	48 (3.91)	36 (4.11)
Moderating variables: Socioeconomic status					
Education	0–16 years, continuous measurement	3.74 (3.89)	3.74 (3.89)	3.97 (3.95)	4.24 (3.96)
Professional prestige before retirement	0–78, continuous measurement	24.39 (12.50)	24.39 (12.50)	24.60 (12.67)	24.64 (12.14)
Annual family income	0–5 million, continuous measurement	15725.885 (22262.464)	17760.867 (39029.371)	15350.826 (35627.239)	19344.679 (36654.534)
Covariates					
Age	60–88 in 2011, continuous measurement	66.63 (5.56)	68.63 (5.56)	70.07 (5.26)	72.22 (4.62)
Gender	Female = 0	624 (41.30)	624 (41.30)	493 (40.15)	332 (37.90)
	Male = 1	887 (58.70)	887 (58.70)	735 (59.85)	544 (62.10)
ADL	Limited = 0	1066 (70.55)	1064 (70.42)	825 (67.18)	592 (67.58)
	Unlimited = 1	445 (29.45)	447 (29.58)	403 (32.82)	284 (32.42)
Area	Rural area = 0	1018 (67.37)	1018 (67.37)	830 (67.59)	601 (68.61)
	Urban area = 1	493 (32.63)	493 (32.63)	398 (32.41)	275 (31.39)
Social activities participation	No = 0	833 (55.13)	740 (48.97)	673 (54.80)	499 (56.96)
	Yes = 1	678 (44.87)	771 (51.03)	555 (45.20)	377 (43.04)
Smoking	No = 0	976 (64.59)	1005 (66.51)	859 (69.95)	579 (66.10)
	Yes = 1	535 (35.41)	506 (33.49)	369 (30.05)	297 (33.90)
Exercise	No = 0	257 (17.01)	261 (17.27)	194 (15.80)	121 (13.81)
	Yes = 1	1254 (82.99)	1250 (82.73)	1034 (84.20)	755 (86.19)
Intergenerational financial support	No = 0	1204 (79.68)	983 (65.06)	783 (63.76)	566 (64.61)
	Yes = 1	307 (20.32)	528 (34.94)	445 (36.24)	310 (35.39)
Intergenerational contact frequency	Seldom or never = 0	738 (48.84)	616 (40.77)	473 (38.52)	309 (35.27)
	Often or sometimes = 1	773 (51.16)	895 (59.23)	755 (61.48)	567 (64.73)
Pension	No = 0	1252 (82.86)	136 (9.00)	228 (18.57)	90 (10.27)
	Yes = 1	259 (17.14)	1375 (91.00)	1000 (81.43)	786 (89.73)
Medical insurance	No = 0	80 (5.29)	39 (2.58)	12 (0.98)	32 (3.65)
	Yes = 1	1431 (94.71)	1472 (97.42)	1216 (99.02)	844 (96.35)

* Note: The samples of the elders not involved in spousal care are deleted here. Only 1383 samples who participated in at least two surveys and in spousal care were retained.

**Table 2:** The operationalization of key concepts of the stress process model.

Stress Process Model	Measures
Stressors: Care Intensity	
Caregivers’ involvement	Duration of care for spouses
Care recipients’ needs	Disability degree of the spouse being cared for
Moderators: Socioeconomic Status	Education level, professional prestige before retirement, and annual family income
Outcome: Depression	CESD-10 Scale

**Table 3:** Depression levels of the older caregivers at different spousal caregiving intensities and disability degree from 2011 to 2018.

		2011		2013		2015		2018	
		Mean (SD)	*p*	Mean (SD)	*p*	Mean (SD)	*p*	Mean (SD)	*p*
Duration of care for spouses	No participation (0 h per week)	9.34(6.38)	0.030	8.11(5.70)	<0.001	8.81(6.82)	0.144	9.11(6.60)	0.053
	Low-level care participation (0.1–9.9 h per week)	10.46(6.67)		9.30(6.32)		9.12(6.42)		9.42(6.52)	
	Moderate-level care participation (10–39.9 h per week)	10.32(6.82)		10.01(6.55)		9.96(7.09)		10.80(7.33)	
	High-level care participation (40–168 h per week)	10.75(7.27)		9.28(6.32)		9.81(7.46)		10.43(7.35)	
Disability degree of the spouse being cared for	No participation	9.34(6.38)	0.001	8.11(5.70)	<0.001	8.81(6.82)	0.006	9.11(6.60)	0.056
	No disability (unlimited ADL but limited IADL) (ADL = 6)	8.83(6.96)		9.88(6.76)		8.17(6.18)		8.71(6.60)	
	Mild disability (7–10)	10.41(6.77)		9.31(6.10)		9.71(7.13)		10.50(7.10)	
	Moderate disability (11–14)	11.73(6.92)		10.43(7.00)		10.22(7.04)		10.50(6.96)	
	Severe disability (15–18)	12.52(6.63)		10.28(6.36)		11.69(7.05)		10.36(7.10)	

**Table 4:** MGM of the effect of spousal caregiving intensity on the depression level of the older caregivers.

	Model 1	Model 2	Model 3	Model 4	Model 5
1. Fixed effects					
Core independent variable					
Duration of care for spouses (Continuous variable)		0.007 ** (0.003)			
Disability degree of the spouse being cared for (Continuous variable)			0.200 *** (0.053)		
Duration of care for spouses (No participation = 0)					
Low level (0.1–9.9 h per week)				0.292 (0.234)	
Moderate level (10–39.9 h per week)				0.931 *** (0.214)	
High level (40–168 h per week)				0.970 ** (0.300)	
Disability degree of the spouse being cared for (No participation = 0)					
No disability (unlimited ADL but limited IADL)					0.262 (0.264)
Mild disability					0.709 ** (0.195)
Moderate disability					1.326 *** (0.342)
Severe disability					1.469 ** (0.486)
Covariates					
Personal characteristics					
Age		0.092 ** (0.035)	0.071 (0.072)	0.085 * (0.035)	0.073 * (0.035)
Gender (female = 0) ^a^		−1.640 *** (0.289)	−1.970 *** (0.515)	−1.627 *** (0.289)	−1.618 *** (0.288)
ADL (unlimited = 0)		2.364 *** (0.184)	3.181 *** (0.364)	2.341 *** (0.184)	2.315 *** (0.185)
Area (rural area = 0) ^a^		−1.190 *** (0.283)	−0.772 (0.506)	−1.180 *** (0.282)	−1.167 *** (0.282)
Health behaviors					
Social activities participation (No = 0)		−0.424 ** (0.162)	−0.310 (0.313)	−0.429 ** (0.162)	−0.428 ** (0.162)
Smoking (No = 0)		0.122 (0.259)	−0.022 (0.477)	0.102 (0.259)	0.105 (0.259)
Exercise (No = 0)		−0.154 (0.250)	0.002 (0.497)	−0.138 (0.250)	−0.148 (0.250)
Intergenerational support					
Intergenerational financial support (No = 0)		−0.539 ** (0.168)	−0.870 * (0.367)	−0.533 ** (0.168)	−0.541 ** (0.168)
Intergenerational contact frequency (Seldom or never = 0)		−0.593 ** (0.194)	−0.769 * (0.375)	−0.590 ** (0.193)	−0.604 ** (0.193)
Social support					
Pension (No = 0)		−0.734 *** (0.164)	−0.779 * (0.340)	−0.762 *** (0.165)	−0.724 *** (0.164)
Medical insurance (No = 0)		−0.019 (0.428)	0.758 (0.928)	−0.060 (0.428)	−0.028 (0.428)
Socioeconomic status					
Education ^a^		−00.131 ** (0.036)	−00.090 (0.064)	−00.130 ** (0.036)	−00.128 ** (0.036)
Professional prestige before retirement ^a^		−00.036 ** (0.011)	−00.051 ** (0.019)	−00.036 ** (0.011)	−00.038 ** (0.011)
Annual family income (natural logarithm)		0.019 (0.052)	−00.060 (0.109)	0.027 (0.052)	0.024 (0.052)
Intercept	9.277 *** (0.137)	12.303 *** (0.684)	11.071 *** (1.480)	12.113 *** (0.685)	12.097 *** (0.685)
2. Random effect					
Intercept SD	4.712 *** (22.205)	4.152 *** (17.235)	4.342 *** (18.856)	4.145 *** (17.182)	4.139 *** (17.127)
Linear slope SD	—	0.501 *** (0.251)	0.654 *** (0.427)	0.502 *** (0.253)	0.505 *** (0.255)
Residual SD	4.500 (20.251)	4.257 (18.122)	4.212 (17.744)	4.250 (18.064)	4.250 (18.060)
ICC	0.512	0.494	0.508	0.494	0.493
Deviance	32282.476	31893.626	8727.002	31870.600	31865.446
N	5126	5126	1383	5126	5126

^a^ Level-2 variables (and the others are level-1 variables); * *p* < 0.05; ** *p* < 0.01; *** *p* < 0.001; standard errors (in parentheses).

**Table 5:** The moderating effect of socioeconomic status.

	Model 6	Model 7
DCS (Continuous variable)	0.013(0.014)	
DDSBC (Continuous variable)		0.616 ***(0.173)
DCS × Education ^a^	−0.001(0.001)	
DCS × Professional prestige before retirement ^a^	0.001(0.001)	
DCS × Annual family income	−0.001(0.002)	
DDSBC × Education ^a^		0.015(0.010)
DDSBC × Professional prestige before retirement ^a^		−0.006 **(0.002)
DDSBC × Annual family income		−0.037 *(0.021)

^a^ Level-2 variables (and the others are level-1 variables); DCS: duration of care for spouses; DDSBC: disability degree of the spouse being cared for; * *p* < 0.10; ** *p* < 0.05; *** *p* < 0.01; standard errors (in parentheses). The covariates included are the same as in Table 4.

## Data Availability

The data that support the findings of this study are openly available in the China Health and Retirement Longitudinal Study at: http://charls.pku.edu.cn/index/en.html (accessed on 20 November 2021).
